# Reducing Depth Measurement Uncertainty in Industrial Robot Stereo Vision Through Error-Aware Disparity Refinement

**DOI:** 10.3390/s26185723

**Published:** 2026-09-09

**Authors:** Lingjiang Meng, Hui Wei, Hongjin Zhang

**Affiliations:** 1 Doctoral Workstation of Precision Visual Perception and Robot Control, School of Artificial Intelligence, Nanning Vocational and Technical University, Nanning 530000, China; 2 School of Computer Science and Technology, Fudan University, Shanghai 200000, China; 3 Laboratory of Algorithms for Cognitive Models and Shanghai Key Laboratory of Data Science, School of Computer Science, Fudan University, Shanghai 200000, China; 4 School of Engineering, Shanghai Ocean University, Shanghai 201306, China; hjzhang@shou.edu.cn

**Keywords:** stereo vision measurement, depth measurement uncertainty, disparity refinement, uncertainty quantification, industrial robot

## Abstract

Stereo vision measurement in industrial robot environments fails on thin structures such as needles and gripper tips, because both traditional and deep-learning-based stereo matching produce boundary errors there. We identify and verify experimentally that ground-truth disparity inflation in public datasets is a systematic error source that pushes networks toward biased boundary estimates. From this, we classify the resulting measurement deviations into four categories: edge errors, intra-segment expansion, small-object loss, and uniform-region distortion. We then propose a geometric-constraint-driven measurement refinement method that corrects each category in turn. Since every correction step is geometric rather than learned, the method is unaffected by ground-truth inflation, a property that conventional post-processing filters do not offer. A GUM-based uncertainty propagation analysis of the measurement model D=f·B/d shows that disparity uncertainty dominates the depth uncertainty budget when *f* and *B* are exactly known. Experiments on KITTI 2015, Middlebury, and a custom UE4 synthetic industrial dataset (100 stereo pairs) with nine stereo baselines (seven deep learning and two traditional) show that, on inflation-free ground truth, the refinement imposes a near-zero systematic penalty on deep learning output while clearly improving traditional methods. On KITTI, the predictable metric shift confirms that the method is unaffected by LiDAR ground-truth inflation. On a real industrial robot scene, the refined disparity recovers the gripper tip and needle that the baseline LEAStereo loses. These results position geometric-constraint-driven refinement as an effective, training-data-independent complement to end-to-end stereo matching for precision industrial measurement, within the tested scenes and methods.

## 1. Introduction

**Background:** Binocular stereo vision recovers three-dimensional structure by triangulating disparity between the left and right views, and it is widely used in industrial robot guidance, precision assembly, and quality inspection. The measurement model is D=f·B/d, where *f* is the focal length, *B* the baseline, and *d* the disparity; these parameters determine the depth accuracy that can be reached [1]. Recent advances continue to push the industrial applicability of binocular measurement through closed-loop calibration for vision-based robots [2], long-range collaborative binocular measurement [3], dual-view stereo sensors for three-dimensional measurement [4], and binocular-based robot calibration [5,6].

**Deep Learning Progress and Industrial Challenges:** Deep stereo matching has evolved from classical cost aggregation [7] to end-to-end CNN-based matching [8], architecture search [9], iterative refinement [10], and Transformer attention [11]. Related efforts include superpixel cost-volume excitation [12], adaptive frequency selection [13], channel attention [14], zero-shot foundation models [15], fusion of monocular priors [16,17], transfer from pretrained vision features [18], and edge-device optimization [19]. On public benchmarks such as KITTI and Middlebury [20,21], these methods now surpass classical approaches [22]. Industrial scenes make different demands on these methods. The precision targets of interest, such as robot gripper tips, thin needles, and metallic workpiece edges, rarely occupy more than a few tens of pixels in the disparity map, yet they need edge localization that generic outdoor benchmarks never exercise. On such scenes, deep stereo methods trained on SceneFlow [23], KITTI [20], and Middlebury [21] show four systematic kinds of local deviation: edge deviation, intra-segment expansion, small-object loss, and uniform-region distortion. They cascade from object boundaries into planar regions and from large to small scales (detailed in Section 2.4).

Table 1 lists recent high-performing stereo matching methods and their reported accuracy on the KITTI 2015 benchmark. These numbers keep improving, yet they average over whole images and do not capture the accuracy on thin structures and object boundaries, which is what industrial measurement requires. Section 2.1 shows this gap on a real industrial scene. Only methods with publicly reported KITTI 2015 D1-all values are listed; the full set of nine baselines used in our experiments is compared in the KITTI and synthetic-dataset experiments of Section 4.

**Limitations:** Two structural causes explain these deviations. First, end-to-end learning never imposes an explicit geometric constraint on measurement accuracy at fine structures. Because the training loss is summed over all pixels, gradients from planar regions, which dominate the pixel count, overwhelm the error signal from thin structures that span at most a few tens of pixels. KITTI makes this worse: its reference disparity maps are interpolated from sparse LiDAR returns and carry inherent edge inflation, so networks trained on it learn biased boundary estimates. Even on Middlebury, where structured-light ground truth has no interpolation inflation, gradual grayscale transitions at object boundaries make deep methods produce interpolated disparity values that belong to neither surface. Second, conventional post-processing, including weighted-median and bilateral filters and segment-based disparity refinement [24], smooths the disparity map without distinguishing structures. It suppresses noise but also erases the needle tips, thin objects, and edges that can be critical in industrial measurement.

**Our Approach:** Instead of redesigning the stereo matching network, we propose a measurement refinement method that is independent of the underlying architecture. The method first diagnoses the four deviation categories in a raw disparity map, then routes each one to a dedicated geometry-driven correction module: edge deviations are resolved by boundary-ownership reassignment; intra-segment expansion is suppressed by piecewise linear fitting; small-object loss is recovered through multi-feature joint matching; and uniform-region distortion is removed by row-column dispersion detection. Every correction step is geometric rather than learned, so the method is unaffected by ground-truth inflation and preserves the fine structures that homogeneous filtering destroys. On the synthetic dataset, KITTI, and Middlebury, we apply the refinement to all nine baseline methods. On real industrial robot images, where no ground truth exists, we apply it to LEAStereo and show the improvement on critical targets such as gripper tips and fine needles.

**The main contributions are summarized as follows (Three-Layer Contribution Framework):**

**Discovery Layer—Theoretical Contribution:** We report an experimental finding. The four deviation classes appear consistently across the nine architectures we tested, persist on datasets with clean ground truth (Middlebury and our synthetic dataset), and are amplified, not caused, by the edge inflation in KITTI ground truth. Three lines of evidence support a structural explanation: the deviations appear on inflation-free ground truth; NAS-searched LEAStereo and iteratively refined IGEV++ show distinct yet consistent failure modes on the same scene; and pixel-aggregated losses apply no optimization pressure toward boundary accuracy.

**Method Layer—Algorithmic Contribution:** We propose the error-aware measurement refinement method described above, where each correction step follows from a diagnosed deviation class rather than from empirical heuristics, and we validate it on nine baselines spanning different architectures.

**Framework Layer—Methodological Contribution:** We put the method inside a validation framework that couples a synthetic industrial dataset with controllable geometric ground truth, uncertainty propagation analysis in line with GUM [25] and VIM [26], and the geometric correction modules. The framework offers a template for industrial measurement tasks in which pixel-level ground truth cannot be obtained.

## 2. Industrial Binocular Measurement: Problem Analysis and Approach

### 2.1. Baseline Assessment on Real Industrial Images

To assess how stereo matching methods perform in real industrial settings, we test two representative deep learning methods on a real industrial robot image: LEAStereo [9] (NAS-based architecture search) and IGEV++ [10] (iterative geometry encoding). The scene contains a gripper with a foam block attached to its end. A thin wire affixed to the foam is too fine for edge detection and barely visible even under magnification. A needle sits on the worktable, separated from the wire by a narrow gap. Together, they represent the two dominant architectural paradigms of current deep stereo matching.

**Figure 1:**
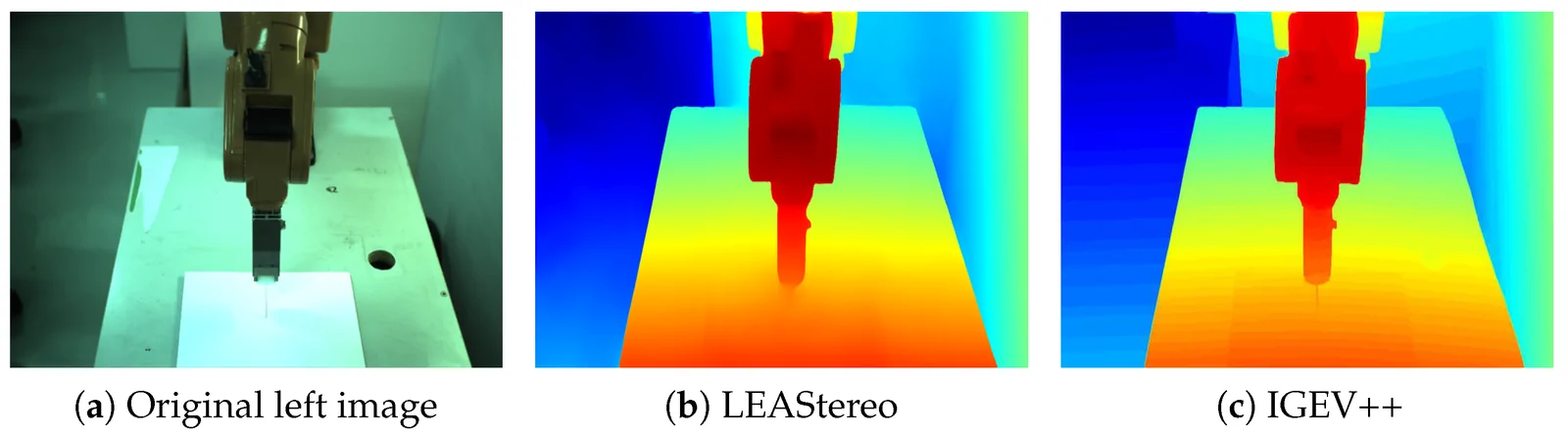
Disparity estimation on a real industrial robot image using two deep learning methods. (**a**) Original left image: a thin wire on the gripper foam tip is separated from the needle by a narrow gap. (**b**) LEAStereo: neither foam nor wire is detected, and the needle tip is submerged by surrounding planar disparity. (**c**) IGEV++: foam and wire are correctly captured, but wire and needle disparities are erroneously fused, eliminating the physical gap.

Figure 1 shows the disparity maps produced by both methods. In all disparity maps, warm colors (e.g., red) denote regions closer to the camera and cool colors (e.g., blue) denote regions farther away; the same color scheme is used throughout the paper. Their failure modes are distinct yet consistent. LEAStereo cannot recover the foam depth because the foam color resembles the white paper on the table and causes severe matching ambiguity. The thin wire is absent as well, and the needle tip region is submerged by surrounding planar disparity. IGEV++ is markedly more sensitive to texture: the foam depth is captured correctly and the thin wire is faintly visible. The wire disparity, however, is fused with the needle disparity, and the physical gap between them disappears.

Taken together, these two failure modes show that higher texture sensitivity does not automatically give better boundary accuracy. Both methods are trained by pixel-aggregated losses, and neither loss enforces an explicit geometric constraint at object boundaries. Whether the network is NAS-searched or iteratively refined, the deficiency persists. Industrial precision targets also differ from generic outdoor scenes in pixel scale and in geometric character, yet existing methods are trained and evaluated only on such outdoor scenes. This motivates building a controllable validation framework in which *f* and *B* are exactly known and the GT disparity is geometrically exact. Pixel-accurate disparity ground truth is extremely difficult to obtain in real industrial scenes, yet deep learning methods need precise supervisory signals to suppress boundary inflation. A natural way out is to build a synthetic industrial dataset in a virtual environment. There, camera parameters, scene geometry, and disparity ground truth are all produced by the rendering engine, which removes the uncertainty of real calibration and gives downstream analysis controlled metrological conditions. That is the subject of the next section.

### 2.2. Synthetic Industrial Dataset with Controllable Geometry

**Figure 2:**
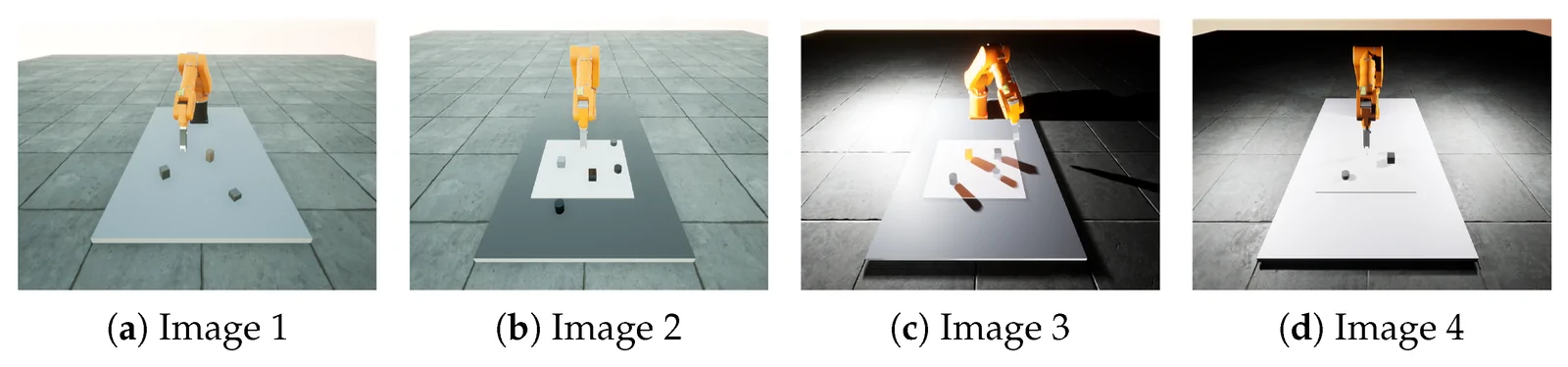
Examples from the synthetic dataset (UE4 industrial robot scenes). (**a**–**d**) Four representative robot-scene pairs rendered in UE4.

Pixel-accurate disparity ground truth is very difficult to obtain in real industrial scenes. LiDAR point clouds are too sparse, structured light is disturbed by specular reflection on metal surfaces, and manual annotation of fine needles introduces error that is hard to control. We therefore adopt the robot manufacturer’s SolidWorks models (SOLIDWORKS 2020 SP05, Dassault Systèmes SolidWorks Corp., Waltham, MA, USA), import them into Unreal Engine 4 (UE4; v4.27, Epic Games, Inc., Cary, NC, USA), and render binocular image pairs [23] while varying the robot pose, lighting, materials, and target objects. Because the original CAD models are used, the geometric dimensions and joint kinematics match the physical equipment exactly. In this virtual environment, camera parameters (focal length *f*, baseline *B*, and so on) are set directly by the CameraActor and can be treated as error-free reference values, so the calibration uncertainty of real cameras is avoided. The dataset contains 100 binocular image pairs. The camera-to-needle working distance is approximately 1.20 m. Ten pairs are selected at uniform intervals along the recorded sequence for evaluation. Images are available at multiple resolutions; evaluation is conducted at 955×579 px. Figure 2 shows examples from the dataset, and Table 2 summarizes the key specifications.

### 2.3. GUM-Based Depth Measurement Uncertainty Analysis

From the binocular measurement model D=f·B/d, the depth measurement uncertainty is given by the GUM law of propagation of uncertainty [25]:

(1) $$\frac{\sigma_{D}}{D}=\sqrt{{\left(\frac{\sigma_{f}}{f}\right)}^{2}+{\left(\frac{\sigma_{B}}{B}\right)}^{2}+{\left(\frac{\sigma_{d}}{d}\right)}^{2}}$$

In this synthetic dataset, f=336 px and B=80 mm are specified directly by the UE4 CameraActor rather than obtained through calibration; hence $\sigma_{f}\approx 0$ and $\sigma_{B}\approx 0$, and the propagation formula simplifies to $\sigma_{D}/D\approx \sigma_{d}/d$, so the disparity term is the sole source of depth uncertainty. The disparity uncertainty $\sigma_{d}$ is evaluated via a Type A method [25] on the reliable measurement regions of the 10 synthetic test images, i.e., over the pixels where disparity estimation is trustworthy, with the regions where it clearly fails (e.g., around depth discontinuities and thin structures) excluded. These excluded failure regions are precisely the target of the selective refinement proposed in this paper. Over the reliable regions we obtain $\sigma_{d}=2.131$ px (bias $\mu_{d}=-0.4$ px). At the working distance of approximately 1.20 m, the combined standard uncertainty is $u_{c}\left(D\right)=114.2$ mm (a relative standard uncertainty of approximately 9.5% of the working distance) and the expanded uncertainty is $U_{D}=228.3$ mm (k=2, approximately 95% confidence level). These values are summarized in Table 3.

This analysis establishes that, in the synthetic data scenario where *f* and *B* are exactly known, the only way to reduce depth measurement uncertainty is to reduce disparity measurement uncertainty. That is the metrological rationale behind the measurement refinement method proposed in this work. In practical industrial deployment, however, *f* and *B* must be obtained through camera calibration, at which point $\sigma_{f}$ and $\sigma_{B}$ should be evaluated from calibration data and included in the full uncertainty budget.

### 2.4. Measurement Error Classification and Correction Rationale

Based on systematic observations on the synthetic dataset, we classify the typical deviations in binocular stereo measurement into four categories, arranged as a causal chain:

- **Edge measurement deviation:** At object boundaries, occlusion between the left and right views makes the boundary disparity belong to neither true surface, producing erroneous cross-boundary assignments.
- **Disparity expansion deviation:** Near occlusion boundaries, disparity from the larger-disparity side extends across the boundary into the smaller-disparity side, introducing disparity jumps within a single object. The two deviations together account for the later loss of small targets.
- **Small-object measurement deviation:** Small targets (e.g., needle tips) have very few matchable pixels. Their matching signal is easily suppressed by edge blur and regional expansion, and the target can disappear from the disparity map.
- **Uniform-region measurement distortion deviation:** Color-homogeneous or texture-poor regions (e.g., a white worktable surface) have strong matching ambiguity and produce irregular wavy distortion of disparity inside them.

These four deviation classes cascade from boundaries to planar regions and from large to small scales, forming a complete causal chain. Existing end-to-end deep learning methods lack a mechanism for diagnosing these deviations and applying geometric-constraint corrections; traditional post-processing filters smooth the disparity map without distinguishing the underlying deviation sources. A measurement refinement method based on error classification and geometric-constraint-driven correction is therefore needed. This is the subject of Section 3.

## 3. Method

Targeting the four classes of measurement deviation identified in Section 2.4, we propose an error-aware, diagnosis-correction framework for measurement refinement. Unlike a generic post-processing filter, the framework first classifies each local deviation into one of four categories, then routes it to a dedicated geometry-driven correction module. This diagnosis-treatment closed loop is what separates it from prior work: boundary ownership reassignment corrects edge deviations, piecewise linear fitting suppresses intra-segment expansion, multi-feature joint matching recovers small-object loss, and row-column dispersion detection removes uniform-region warping. Each module follows geometric constraints rather than data-driven learning, so the framework is not biased by GT inflation in the training data.

**Prerequisite and applicability boundary.** The geometric constraints of this method rely on Canny edge detection as a prerequisite. When edges are detectable, the boundary ownership reassignment module can successfully separate objects that are fused in the disparity map (e.g., the wire-needle fusion produced by IGEV++). The limitation arises when the target and background are similar in color such that edges are undetectable (e.g., white foam against white paper). In such cases, the affected regions are treated as background by subsequent modules and their disparity cannot be recovered. This represents the fundamental difference between purely geometry-driven approaches and texture-sensitive deep learning methods.

For clear visualization of each correction module’s effect, the following sections use LEAStereo [9] as the demonstration baseline. As Section 2.1 shows, LEAStereo exhibits the most typical disparity defects (needle tip submerged by planar disparity), making the effect of each correction module most visually discernible. The figure below shows the complete refinement pipeline.

**Figure 3:**
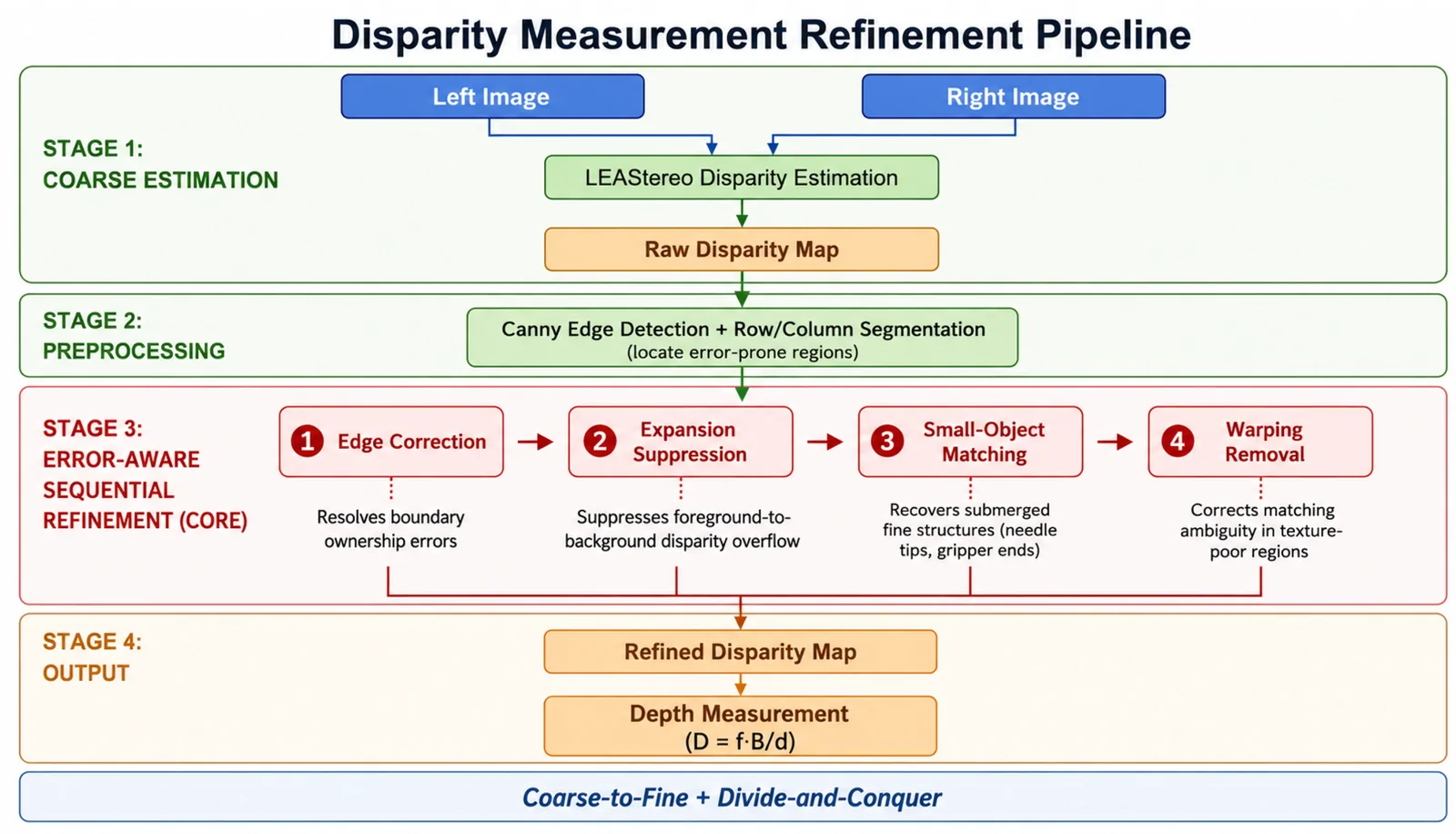
Disparity measurement refinement pipeline. Four sequential stages: ① Edge correction → ② Intra-segment expansion suppression → ③ Small-object matching recovery → ④ Uniform-region warping removal.

As shown in Figure 3, the measurement refinement pipeline has four stages.

**Stage 1: Coarse estimation.** The left and right stereo images are first passed through a bilateral filter to suppress noise, then fed into LEAStereo, which performs end-to-end stereo matching to produce a raw disparity map.

**Stage 2: Preprocessing.** Canny edge detection is applied to the raw disparity map, combined with row- and column-wise pixel segmentation, to identify regions prone to measurement deviations, including object boundaries and texture-ambiguous areas.

**Stage 3: Error-aware sequential refinement (core).** Four geometric-constraint modules execute in the causal-chain order established in Section 2.4: ① Edge correction: resolving boundary-ownership errors and larger/smaller disparity confusion; ② Intra-segment expansion suppression: eliminating larger-disparity spill-over into smaller-disparity regions; ③ Small-object matching recovery: retrieving submerged fine structures such as needle and gripper ends; ④ Uniform-region warping removal: correcting deformation caused by matching ambiguity in texture-poor areas. The output of each step serves as the input to the next, forming a cascaded correction chain that ensures each class of deviation is handled independently and thoroughly.

**Stage 4: Output.** The refined disparity map, combined with the known camera parameters *f* and *B*, is converted to depth measurements via the binocular model D=f·B/d, which gives the final measurement result. A single-point failure in any earlier module does not halt the pipeline, as each downstream module operates on the output of its predecessor; this sequential architecture provides fault tolerance by construction.

**Figure 4:**
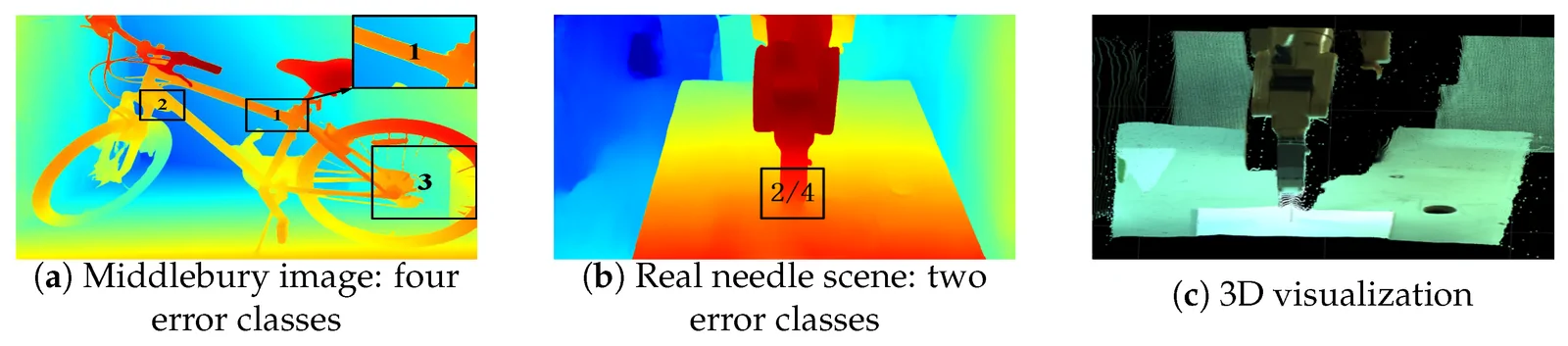
Visual diagnosis of the four measurement deviation classes. (**a**) Middlebury image: four error classes; (**b**) Real needle scene: two error classes; (**c**) 3D visualization.

Building on the theoretical classification in Section 2.4, Figure 4 visualizes the four systematic measurement deviation classes as they appear in LEAStereo disparity maps. Figure 4a selects a representative Middlebury test image and labels all four deviation classes: edge deviation (disparity at boundaries belonging to neither true surface), intra-segment expansion (larger-disparity values spilling across boundaries into smaller-disparity regions), small-object loss, and uniform-region distortion. Figure 4b annotates two deviation classes on a real industrial needle image: small-object loss in the needle region and disparity expansion around the needle. Figure 4c presents a 3D visualization of the same regions with disparity mapped to depth, where the deviation classes manifest as abnormal protrusions, depressions, or undulations. The needle tip, which should appear as an independent thin line in 3D, is completely erased by the combined effect of small-object loss and planar-region distortion. Together, these three subfigures form a complete measurement deviation diagnostic atlas, providing clear visual targets for the design of each subsequent correction module.

### 3.1. Edge Disparity Problem

**Figure 5:**
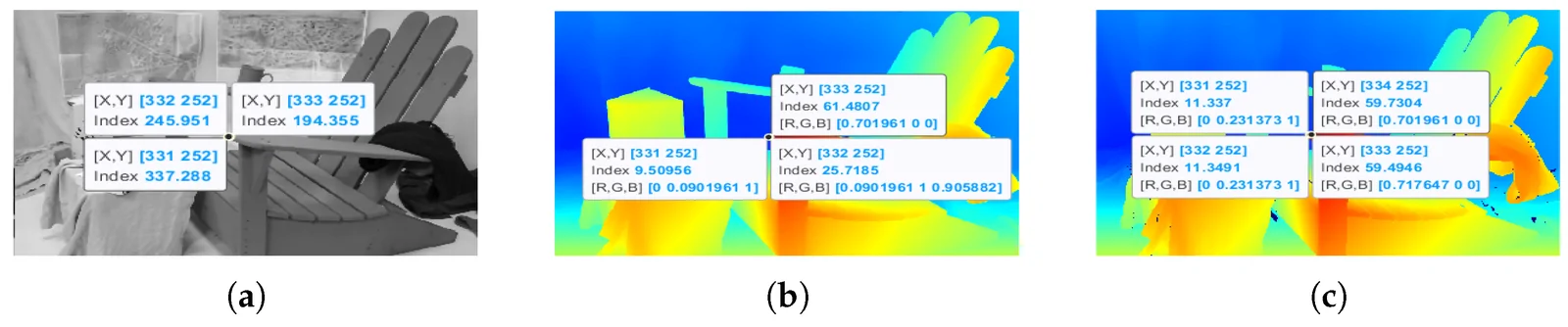
Edge disparity measurement deviation. (**a**) Grayscale: sampled intensity across edge; (**b**) LEAStereo: interpolated disparity at edge; (**c**) GT: sharp disparity step at edge.

**Edge Disparity Deviation:** Figure 5 illustrates the first deviation class through three subfigures. Figure 5a shows the grayscale image with an intensity profile sampled across an object junction. Instead of a sharp step, the intensity transitions gradually over several pixels, an inherent imaging artifact rather than the true geometric boundary. Figure 5b shows the LEAStereo disparity estimate at the same locations. The disparity also transitions gradually at the boundary, which shows that LEAStereo inherits the edge blur from the grayscale image and produces interpolated disparity values belonging to neither surface. Figure 5c shows the reference (GT) disparity, where the sampled points form a sharp step: the disparity belongs to one side or the other, with no intermediate values. The three panels together show that deep learning methods produce interpolated disparity that co-varies with the grayscale gradient and deviates from the true step boundary. This is the first class of systematic measurement deviation. Our correction strategy replaces the affected boundary disparities with those of the occluded side, the smaller-disparity side.

### 3.2. Disparity Expansion

**Figure 6:**
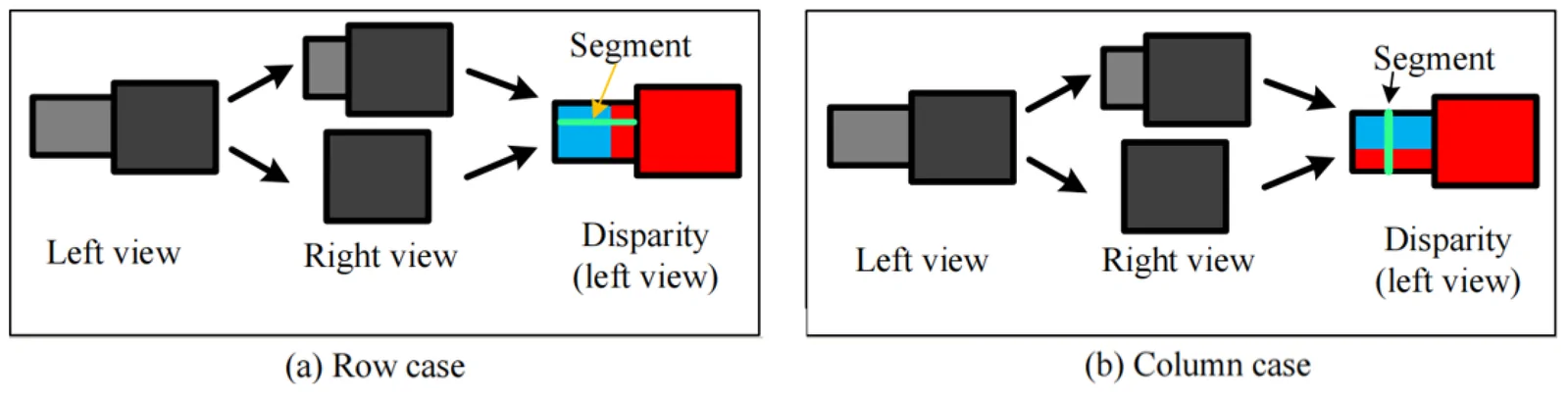
Intra-segment disparity expansion.

**Disparity Expansion:** It extends disparity from an occluding object (larger disparity) into the region of the occluded object (smaller disparity), producing a disparity jump within the pixel segment on the occluded side. Rows and columns are processed separately. With the image disparity denoted dis(x,y), the row-wise and column-wise jump conditions are given by Equation (2) and illustrated in Figure 6a. The larger-disparity and smaller-disparity sides of the jump form two candidate regions.

(2) $$\left\{\begin{array}{c} |\mathrm{dis}(x,y-1)-\mathrm{dis}(x,y)|>\mathrm{threshold}\\ |\mathrm{dis}(x,y)-\mathrm{dis}(x,y+1)|>\mathrm{threshold}\\ |\mathrm{dis}(x,y-3)-\mathrm{dis}(x,y+3)|>\mathrm{threshold} \end{array}\right.,\;\left\{\begin{array}{c} |\mathrm{dis}(x-1,y)-\mathrm{dis}(x,y)|>\mathrm{threshold}\\ |\mathrm{dis}(x,y)-\mathrm{dis}(x+1,y)|>\mathrm{threshold}\\ |\mathrm{dis}(x-3,y)-\mathrm{dis}(x+3,y)|>\mathrm{threshold} \end{array}\right.$$

The row case is processed first, as illustrated in Figure 6a: the gray box marks the occluded side (smaller disparity), the black box the occluding side (larger disparity), and blue and red denote smaller and larger disparity values, respectively. In this schematic, the right view may be partially or fully occluded, which does not affect the row-wise expansion processing for the left view. The smaller-disparity side is then selected and linearly fitted, and the fitted values fill the entire segment.

For the column case, illustrated in Figure 6b, pixels within the same column segment are first grouped by disparity. The group with the most elements and a correct disparity is then linearly fitted and used to replace the other positions in that segment. The correctness of the intra-group disparity is verified by tracking the correspondence from the left image to the right image through the disparity.

The validity of this rule presupposes substantially continuous disparity within each segment. When Canny edge detection successfully separates depth layers, each segment contains a single dominant disparity and the fitting-and-replacement rule is reliable. Two scenarios can cause failure. In the first, multiple depth layers coexist within one segment. When upstream edge detection misses a depth boundary (for example, in weak-texture or low-contrast regions), a single segment may contain several unresolved depth layers, and selecting the smaller-disparity side is then inapplicable. This limitation comes from the method’s reliance on edge detection; adjusting the detection thresholds mitigates it but does not eliminate it. In the second scenario, boundaries are irregular, tilted, or curved. Row- and column-wise decomposition has difficulty with strongly tilted or curved occlusion boundaries. In practical industrial scenes, for example, desktop edges, object contours, and robot joints, most occlusion boundaries are roughly horizontal or vertical. At pixel-level discretization, any curved boundary is built from row-wise and column-wise pixel steps, so the row/column approximation is naturally justified. Under these conditions, the simplification is a reasonable engineering trade-off.

This module involves two core parameters: segment_threshold = 2, controlling the minimum number of pixels required for a valid row/column segment, and segmentDisparity_threshold = 3, controlling the minimum adjacent-pixel disparity difference that qualifies as a segment conflict. These values are typical for KITTI scenes and should be adjusted when switching to other scenarios.

### 3.3. Disparity of Tiny Objects

**Figure 7:**
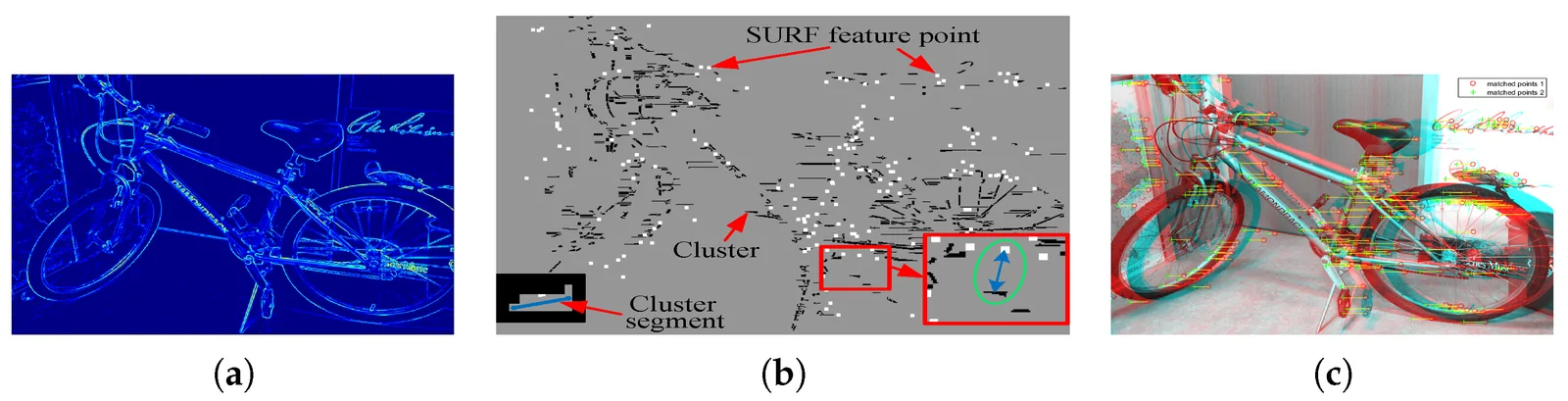
Small-target matching elements. (**a**) Gradient image; (**b**) Matching unit; (**c**) SURF feature-point matching.

A small object such as the needle or the gripper tip often has no reliable match in the right image, so its pixels are left with a disparity of 0 and appear as black regions in the disparity map. These zero-disparity pixels are exactly the regions the method must recover. Direct pixel-based matching is unreliable here, because the object is too small and too weakly textured to disambiguate a match. The method therefore adds two auxiliary cues: the gradient image (Figure 7a) and SURF [27] feature points. Zero-disparity pixels that touch each other are grouped into one cluster. Such a connected component may extend along a row, along a column, or along an inclined direction. The first and last pixels of each cluster are joined into a straight line, which is called a cluster segment and serves as a linear approximation of the component, as illustrated in Figure 7b. For each cluster segment, the SURF feature point whose intensity is closest to the intensity of the segment centroid (white dots in Figure 7c) is chosen as the matching anchor; the anchor and the cluster segment together form a matching unit (green circles). The matching unit supplies three cues for disparity recovery: the intensity of every pixel in the cluster segment, the gradient of every pixel in the cluster segment, and the intensity difference between the SURF anchor and the cluster centroid.

SURF matching quality is controlled by a threshold, profile_score_threshold = 0.4. A candidate match is kept only if its SURF score reaches 0.4; weaker matches are discarded. When no candidate survives, the algorithm falls back to the regional median or to neighborhood extrapolation. The value 0.4 was calibrated on representative KITTI scenes; because it is expressed in pixel units, it scales with the image resolution and the disparity range of the scene and should be adjusted when the method moves to other scenarios.

**Figure 8:**
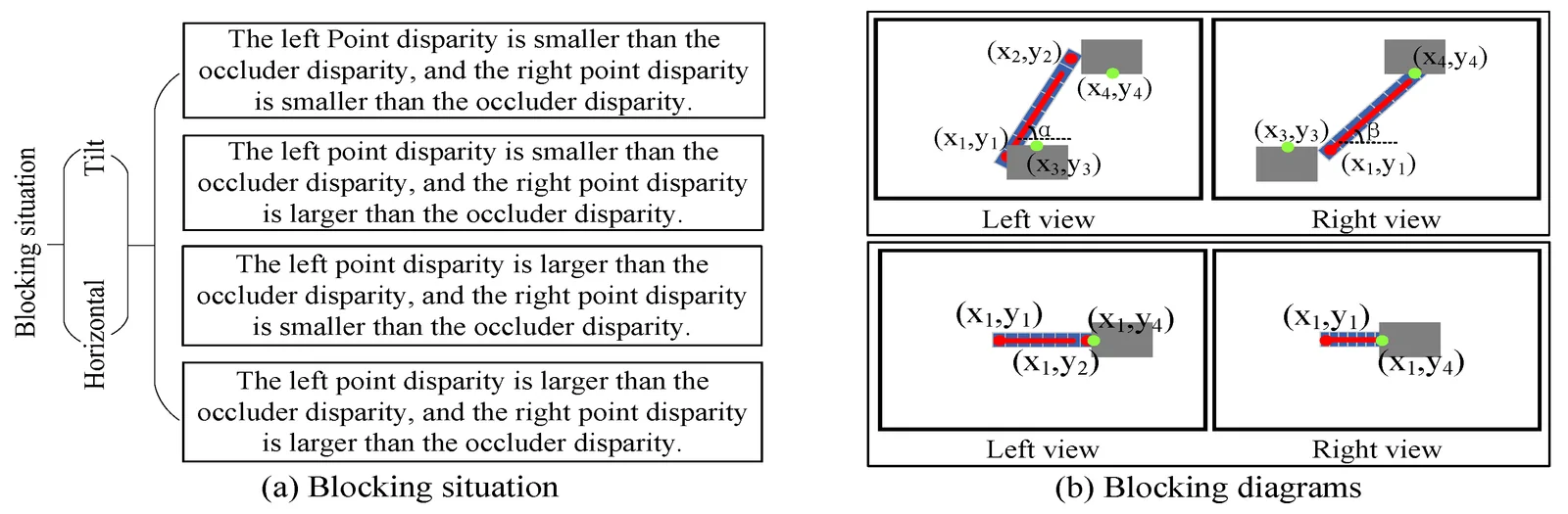
Schematic of the occlusion (blocking) situation. (**a**) Blocking situation; (**b**) Blocking diagrams.

When establishing correspondences, a cluster segment may be partially occluded. If a cluster segment is obscured when the left and right views are aligned, the unobscured portion of the cluster segment is used for matching instead, as illustrated in Figure 8.

When the cluster segment is tilted, the coordinates of the lower and upper endpoints are $\left(x_{1},y_{1}\right)$ and $\left(x_{2},y_{2}\right)$. The straight-line equation is

$$\frac{y-y_{1}}{y_{2}-y_{1}}=\frac{x-x_{1}}{x_{2}-x_{1}},$$

and the coordinates of the lower and upper endpoints of the unblocked segment part are then

(3) $$\left\{\begin{array}{c} \left(x_{d},y_{d}\right)=\left(x_{1},y_{1}\right),\;D_{\left(x_{1},y_{1}\right)}>D_{\left(x_{3},y_{3}\right)}\\ \left(x_{d},y_{d}\right)=\left(x_{3},\frac{\left(x_{3}-x_{1}\right)\left(y_{2}-y_{1}\right)}{x_{2}-x_{1}}+y_{1}\right),\;D_{\left(x_{1},y_{1}\right)}\leq D_{\left(x_{3},y_{3}\right)} \end{array}\right.$$

and

(4) $$\left\{\begin{array}{c} \left(x_{u},y_{u}\right)=\left(x_{2},y_{2}\right),\;D_{\left(x_{2},y_{2}\right)}>D_{\left(x_{4},y_{4}\right)}\\ \left(x_{u},y_{u}\right)=\left(x_{4},\frac{\left(x_{4}-x_{1}\right)\left(y_{2}-y_{1}\right)}{x_{2}-x_{1}}+y_{1}\right),\;D_{\left(x_{2},y_{2}\right)}\leq D_{\left(x_{4},y_{4}\right)} \end{array}\right.$$

respectively, where $x_{d}$ denotes the lower endpoint row coordinate, $y_{d}$ the lower endpoint column coordinate, $x_{u}$ the upper endpoint row coordinate, and $y_{u}$ the upper endpoint column coordinate. $D(x_{n},y_{n})$ represents the disparity of the point.

In Equations (3) and (4), $\left(x_{3},y_{3}\right)$ and $\left(x_{4},y_{4}\right)$ are points on the occlusion boundary (the gray occluder in Figure 8) on the lower and upper sides of the segment, respectively. Their disparity values $D_{\left(x_{3},y_{3}\right)}$ and $D_{\left(x_{4},y_{4}\right)}$ are compared with those of the segment endpoints to decide which part of the segment is occluded: an endpoint whose disparity exceeds the occluder disparity is visible, so the unoccluded segment starts (or ends) at that endpoint; otherwise the endpoint is occluded and the unoccluded segment resumes at the occlusion boundary, whose row coordinate is $x_{3}$ (or $x_{4}$), with the column coordinate obtained from the straight-line equation of the segment.

When the cluster segment is horizontal, it lies along a fixed row, so the left and right endpoints are $\left(x_{1},y_{1}\right)$ and $\left(x_{1},y_{2}\right)$ (i.e., $x_{2}=x_{1}$), and the occluder point on the right side is $\left(x_{1},y_{4}\right)$. They satisfy the following condition:

(5) $$\left(x_{l},y_{l}\right)=\left(x_{1},y_{1}\right),\;\left\{\begin{array}{c} \left(x_{r},y_{r}\right)=\left(x_{1},y_{4}\right),\;D_{\left(x_{1},y_{2}\right)}<D_{\left(x_{1},y_{4}\right)}\\ \left(x_{r},y_{r}\right)=\left(x_{1},y_{2}\right),\;D_{\left(x_{1},y_{2}\right)}\geq D_{\left(x_{1},y_{4}\right)} \end{array}\right.$$

Once the coordinates of the two endpoints of the cluster segment are determined, the pixel coordinates within the segment can be calculated using the linear equation of the cluster segment.

The three cues of the matching unit are combined into a weighted matching score. Let the cluster involved in matching contain *n* pixels. We first sum each cue over the *n* pixels. The summed intensity, summed gradient, and summed feature-point difference are given by:

(6) $$\begin{array}{cccc} S_{\mathrm{gray}} & =\sum_{1}^{n}\mathrm{Gray}\left(x_{n},y_{n}\right),S_{\mathrm{gradient}} & =\sum_{1}^{n}\mathrm{Gradient}\left(x_{n},y_{n}\right),S_{gf} & =\sum_{1}^{n}\left(\mathrm{Gray}\left(x_{n},y_{n}\right)-\mathrm{Gray}\left(x_{f},y_{f}\right)\right), \end{array}$$

where $\left(x_{f},y_{f}\right)$ satisfies condition

(7) $$\begin{array}{ccc} \; & \mathrm{Gray}\left(x_{f},y_{f}\right)-\mathrm{Gray}\left(x_{\mathrm{mid}},y_{\mathrm{mid}}\right)=\; & \min\left\{\mathrm{Gray}\left(x_{k},y_{k}\right)-\mathrm{Gray}\left(x_{\mathrm{mid}},y_{\mathrm{mid}}\right)|k=1,2,\ldots ,m\right\}, \end{array}$$

where $\left(x_{mid},y_{mid}\right)$ is the centroid of the cluster segment and *m* is the number of SURF feature points. Each summed cue is then normalized into a weight, so that the three cues are comparable in magnitude:

(8) $$\begin{array}{cccc} W_{\mathrm{gray}} & =\frac{S_{\mathrm{gray}}+S_{\mathrm{gradient}}+S_{gf}}{3S_{\mathrm{gray}}},W_{\mathrm{gradient}} & =\frac{S_{\mathrm{gray}}+S_{\mathrm{gradient}}+S_{gf}}{3S_{\mathrm{gradient}}},W_{gf} & =\frac{S_{\mathrm{gray}}+S_{\mathrm{gradient}}+S_{gf}}{3S_{gf}}. \end{array}$$

For a tilted cluster segment, the viewpoint change from the left to the right image also changes the apparent tilt angle of the segment. This angle is unknown before the disparity is computed, so the method evaluates the matching score over a range of candidate angles in the right image. The tilt angle is searched over the range from 0 to 180 degrees. Let α be the tilt angle of the cluster segment in the left image and β be a candidate tilt angle in the right image. For a cluster of *s* pixels, the row and column coordinates of the *t*-th pixel (t=1,2,…,s) at these two angles are related by

(9) $$y_{t}{|}_{x_{t}}=\tan\alpha \cdot |x_{d}-x_{u}|,\;y_{t^{\Delta }}{|}_{x_{t}}=\tan\beta \cdot \left|x_{d}-x_{u}\right|,\;t\in \left\{1,2,\ldots ,s\right\},$$

where $x_{t}$ and $y_{t}$ are the row and column coordinates of the *t*-th pixel of the segment in the left image, and $y_{t^{\Delta }}$ is its column coordinate in the right image at the candidate angle β. For a horizontal cluster segment, a change of viewpoint shortens or elongates it. Based on practical experience, the method allows the length to shrink to half of the original or to grow to twice the original length. This range is captured by a length factor θ, which lies in [1/2,2], and the column coordinate of the *t*-th pixel in the right image is $y_{t^{\Delta }}{|}_{x_{t}}=y_{t}\cdot \theta$.

The final matching score combines the three normalized weights with the per-pixel cue differences between the two views. The candidate angle that yields the smallest score is taken as the correct match, and the resulting disparity is assigned to all pixels of the cluster segment.

(10) $$W_{\mathrm{gray}}\cdot \sum_{1}^{s}\left|Gy\right|+W_{\mathrm{gradient}}\cdot \sum_{1}^{s}\left|Gd\right|+W_{gf}\cdot \sum_{1}^{s}\left(Gs\right)$$

where $Gy_{t}=Gray\left(x_{t},y_{t^{\Delta }}\right)-Gray\left(x_{t},y_{t}\right)$ and $Gd_{t}=Gradient\left(x_{t},y_{t^{\Delta }}\right)-Gradient\left(x_{t},y_{t}\right)$ are the gray and gradient differences between the two views at the *t*-th pixel, and $Gs_{t}=\left(Gray\left(x_{t},y_{t}\right)-Gray\left(x_{f},y_{f}\right)\right)-\left(Gray\left(x_{t},y_{t}\right)-Gray\left(x_{f^{\Delta }},y_{f^{\Delta }}\right)\right)$ measures the change of the anchor feature-point distance between the left image, where the anchor is at $\left(x_{f},y_{f}\right)$, and the right image, where the corresponding anchor is at $\left(x_{f^{\Delta }},y_{f^{\Delta }}\right)$.

**Figure 9:**
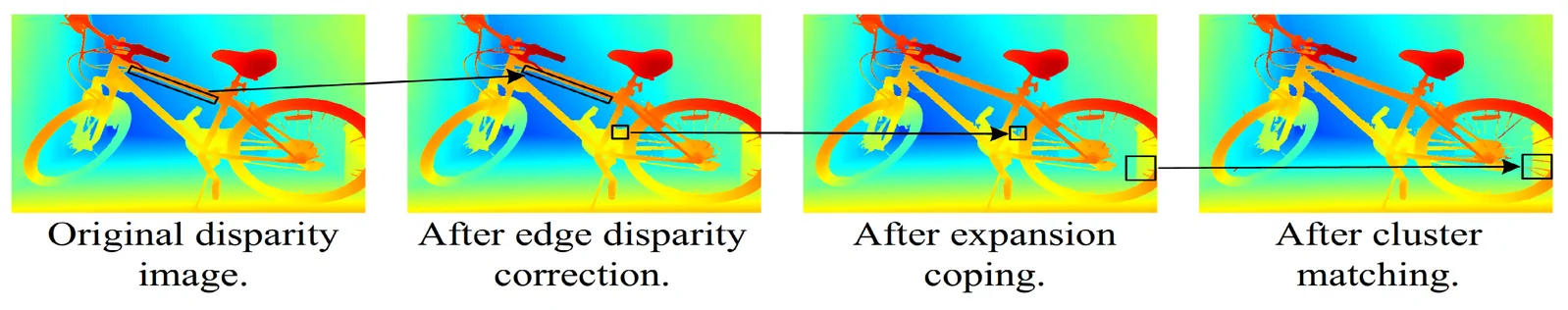
Measurement refinement results on the synthetic needle image.

If a cluster segment still has no disparity after score-based matching, the method extrapolates from the region just outside the segment ends. It searches for a pixel outside the segment whose color is close to the segment and whose disparity is greater than 0, and takes the larger candidate value as the disparity for the whole cluster. As a second fallback, the method scans the original disparity map at the zero-disparity pixels inside the segment and fills them with the maximum value found. Figure 9 shows the results of these steps and marks where each step improved the map.

### 3.4. Disparity Warping Variation

**Figure 10:**
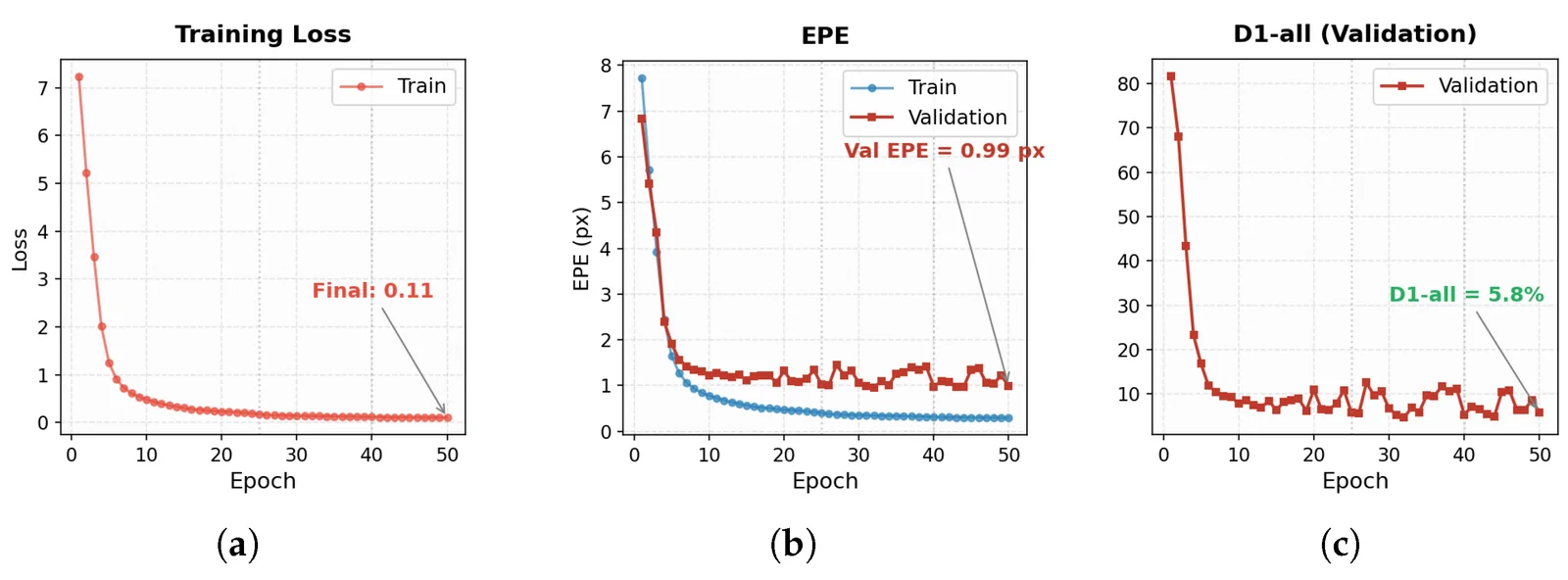
Domain adaptation training curves of LEAStereo on the synthetic dataset. (**a**) Training loss; (**b**) EPE (train/val); (**c**) D1-all (val).

The disparity warping addressed in this subsection occurs in color-homogeneous pixel segments, meaning weak-texture regions such as a white worktable surface, where matching ambiguity produces irregular disparity undulations. To see both the potential and the limits of domain-adaptive fine-tuning for correcting such warping, we fine-tuned LEAStereo on the UE4 synthetic industrial dataset described in Section 2.2. Figure 10 shows the full training curves. Training and validation metrics converge smoothly with no overfitting, and the validation EPE reaches about 0.99 px. Yet even after full convergence, the fine-tuned disparity map still fails to recover the needle tip and the other small structures. This suggests that adding training data does not compensate for the structural limits of the end-to-end learning architecture; the root cause is in the architecture itself, not in data quantity. All deep stereo methods in our quantitative experiments therefore use their originally published pretrained models for inference, which keeps the comparison fair and consistent.

**Figure 11:**
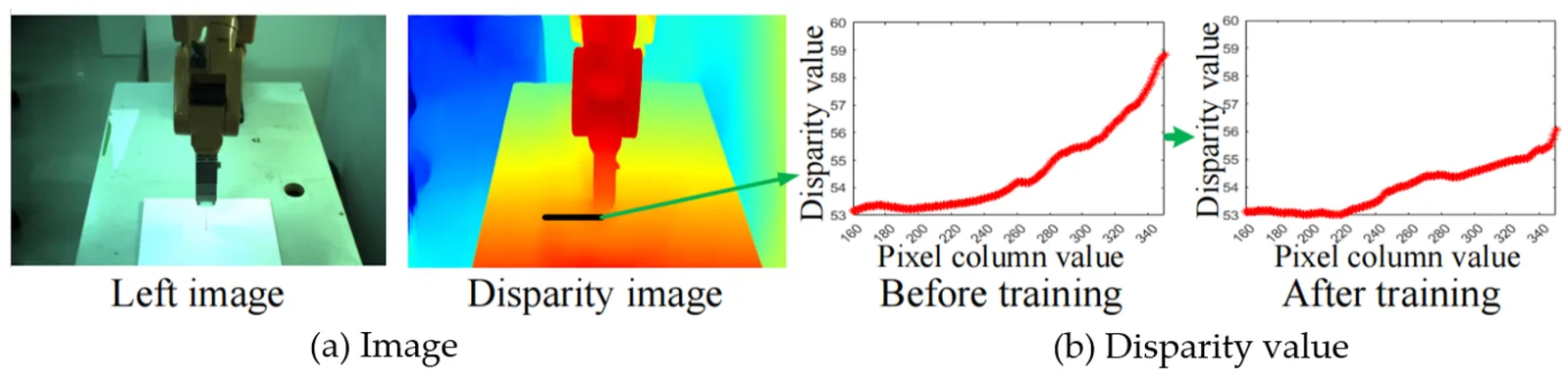
Disparity warping problem before and after fine-tuning.

The processing results after training are shown in Figure 11. As seen from the figure, while disparity warping has improved after training, it is still insufficient to fully resolve the issue. Further processing is needed.

For each uniform-color region, the per-row disparity dispersion is computed as

(11) $$s^{2}=\frac{\sum_{1}^{n}{\left(\bar{x}-x_{n}\right)}^{2}}{n}.$$

**Figure 12:**
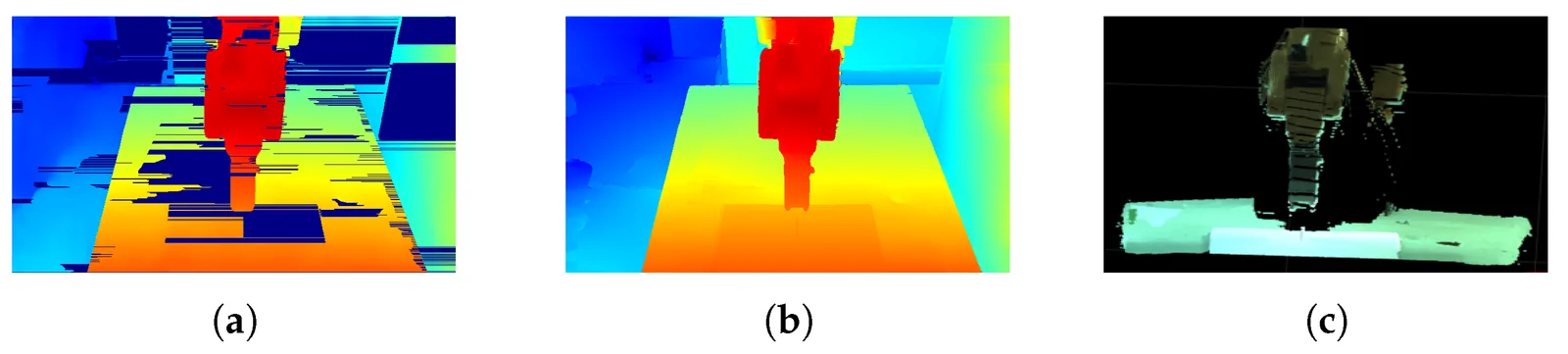
Removal of high-dispersion regions and final refinement results. (**a**) Removes areas of large disparity dispersion; (**b**) The results after the fitting process; (**c**) The final result corresponds to 3D map.

Rows whose dispersion exceeds the median dispersion across all rows in the region are treated as distorted and removed entirely. The removed disparities are then reconstructed by linear fitting along both the row and column directions. This process is fully automatic. Figure 12 displays the result of removing the high-dispersion region disparity, the final result after fitting, and the corresponding 3D map. The needle in the final image is clearly identifiable, and the disparity results in other areas are also accurate.

## 4. Experiment and Result

We compare the proposed measurement refinement method against nine baselines covering both traditional and deep learning approaches. The traditional methods are ELAS [28], which accelerates large-scale reconstruction through line-segment matching, and SGM [7], which obtains per-pixel matching through semi-global mutual information optimization. The deep learning baselines span the main architectural families. FC-DCNN [29] uses dense convolutional connections for lightweight matching; FCDSN-DC [30] augments CNN matching with depth completion; GANet [8] learns semi-global cost optimization through guided aggregation; MaDis-Stereo [11] applies Transformer attention with masked image modeling distillation for cross-scene robustness; LEAStereo [9] searches optimal network structures through hierarchical neural architecture search; IGEV++ [10] builds multi-range geometry encoding volumes for iterative refinement; and DEFOM-Stereo [18] transfers pretrained vision foundation models to stereo matching. Together they cover CNN, NAS, Transformer, iterative refinement, and foundation model transfer, which are the mainstream technical routes in contemporary stereo matching.

### 4.1. KITTI Evaluation

**Figure 13:**
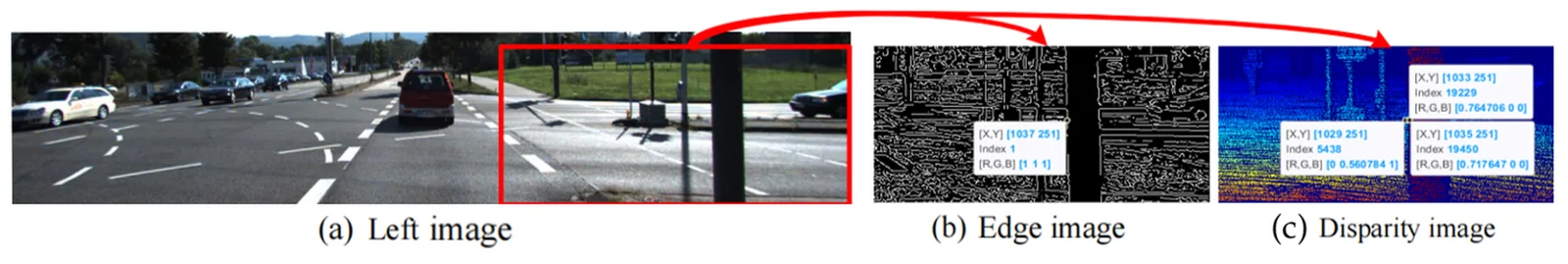
Edge inflation in KITTI LiDAR ground truth. (**a**) Left image; (**b**) Edge image; (**c**) Disparity image, where tree disparity extends beyond the tree-trunk boundary.

**KITTI Experiment Rationale:** Unlike the Middlebury dataset, the KITTI 2015 ground truth [20] has inherent edge disparity inflation. Its GT is generated by interpolating sparse LiDAR point clouds and projecting them onto the image plane, so disparity values at object boundaries expand beyond the true edges (Figure 13, where tree disparity extends beyond the detected edge boundary). This inflated GT conflicts with our edge correction module in a predictable way. When the algorithm shrinks inflated disparity edges back to their correct positions, comparison with the inflated GT classifies these corrections as “errors,” which shows up as an apparent increase in the evaluation metrics. We still run quantitative evaluation on the KITTI 2015 training set, for a precise reason. Because this conflict is predictable, the metric shift should be highly consistent in magnitude across all methods and architectures. This makes KITTI a natural controlled experiment for verifying that our method is insensitive to GT inflation.

**KITTI as a Diagnostic Benchmark:** We use KITTI 2015 not to validate the proposed method but to check how reliable its LiDAR-derived ground truth is for boundary-precision evaluation. We select 10 uniformly spaced images from the KITTI 2015 training set and apply measurement refinement to all nine stereo matching methods. Results are reported in Table 4.

**Diagnostic Finding:** Two observations emerge from Table 4. First, all seven deep learning methods show consistent metric degradation after refinement: D1-all increases by +0.35 to +0.60 pp, regardless of the method’s own accuracy level (GANet at 0.34% and FCDSN-DC at 8.84% shift by comparable magnitudes). This cross-architecture consistency is the signature of ground-truth inflation rather than method-specific error. Every deep stereo network tested was trained on SceneFlow and KITTI, so it learned to predict the inflated LiDAR-derived boundary disparity as ground truth. When our boundary correction shrinks inflated edges back to geometrically correct positions, the comparison against inflated GT registers a systematic deviation. Second, traditional methods (ELAS, SGM) improve sharply (D1-all reductions of 26.84 and 15.65 pp), because their errors are concrete defects, such as voids and expansion, rather than learned biases, and our geometric corrections address those defects directly.

To confirm that these averages are not driven by individual images, we quantify the per-image spread of each Δ metric across the 10 KITTI images. For the seven deep-learning methods, ΔD1-all lies between +0.35 and +0.60 pp with a per-image standard deviation of only 0.12–0.25 pp, and the per-image change is positive for every image of every method, without a single exception. For ELAS and SGM, the improvement is −26.84±3.96 pp and −15.65±3.05 pp, respectively, again consistently negative across all 10 images. The degradation (resp. improvement) is therefore a systematic, image-independent effect rather than an artifact of averaging over a few outlier images.

**Implication for Benchmark Practice:** The consistent degradation of all deep learning methods on inflated KITTI GT points to a concrete limitation: inflated boundary GT makes KITTI unreliable for assessing boundary measurement accuracy, no matter which deep stereo method is used. This is why we rely on Middlebury (structured-light GT, free of interpolation inflation) and the UE4 synthetic dataset (geometrically rendered GT) as the primary evaluation benchmarks.

**Method Selection for Industrial Experiments:** Based on the above analysis, we select LEAStereo as the baseline for the industrial robot experiments for two reasons. First, it comes from the NAS-search family and is not saturated on our inflation-free evaluation datasets. Second, its output shows the most visually discernible deviation patterns on fine industrial structures, so it is a demanding baseline that exposes the effect of each correction module.

The measurement refinement framework takes about 1.2 s per image (resolution 591×992) in MATLAB R2021a on an AMD Ryzen 7 5700X CPU with an NVIDIA GeForce RTX 3060 GPU and 32 GB system RAM, which is acceptable for offline analysis. For latency-sensitive applications, MEX compilation and a C++ reimplementation could reduce the runtime substantially.

### 4.2. Industrial Robot Scenario

#### 4.2.1. Synthetic Dataset

**Synthetic Dataset Evaluation:** The synthetic UE4 dataset (10 images, dense GT, free of edge inflation) provides a geometrically exact ground truth, serving as a critical control for verifying the GT-inflation immunity of our method. As shown in Table 5, the deep learning methods (MaDis, GANet, IGEV++, LEAStereo, DEFOM, FC-DCNN, FCDSN-DC) all show near-zero metric changes before and after refinement: ΔBad4, ΔEPE, and ΔD1 each fall within ±0.1, and ΔBad1 within ±0.12 (the largest deviation, ΔBad1 = +0.12 pp, belongs to IGEV++), with some methods improving slightly and others degrading slightly, with no systematic direction. In contrast, the traditional methods ELAS and SGM gain substantially: ΔBad4 = −5.98 and −4.98 pp; ΔEPE = −4.52 and −3.80 px. This comparison clarifies the picture: when GT is inflation-free, measurement refinement imposes no systematic penalty on already accurate deep learning methods, while retaining its effectiveness on traditional methods with larger initial errors, in direct contrast to the apparent degradation observed on KITTI. Per-image variability is even smaller on this dataset: for the seven deep-learning methods, the standard deviation of ΔD1-all across the 10 images is at most 0.05 pp. The near-zero means in Table 5 therefore do not arise from cancellation between large positive and negative per-image changes; they reflect genuinely unchanged performance on every single image, which confirms that refinement neither helps nor harms accurate deep-learning predictions on inflation-free GT.

Table 5 also links the disparity-domain metrics to the GUM depth uncertainty analysis of Table 3. The EPE in the table and the disparity uncertainty $\sigma_{d}$ in the budget are both pixel-domain measures of disparity error, so a change in EPE signals a change of the same sign in $\sigma_{d}$. For the deep learning methods, whose initial disparity is already accurate, refinement leaves EPE almost unchanged (for example, LEAStereo: 6.88→6.89 px, ΔEPE =+0.01 px). Their $\sigma_{d}$, and hence the depth uncertainty $u_{c}\left(D\right)=114.2$ mm, stay almost unchanged, which confirms that refinement does not degrade the metrological accuracy of an already accurate method. For the traditional methods, refinement lowers EPE markedly (ELAS: 21.4→16.9 px, about −21%; SGM: 21.0→17.2 px, about −18%). Under the GUM propagation formula $\sigma_{D}/D\approx \sigma_{d}/d$, a disparity-error reduction of this magnitude lowers the relative depth uncertainty by the same proportion, so refinement directly improves the measurement uncertainty of the final depth values.

**Figure 14:**
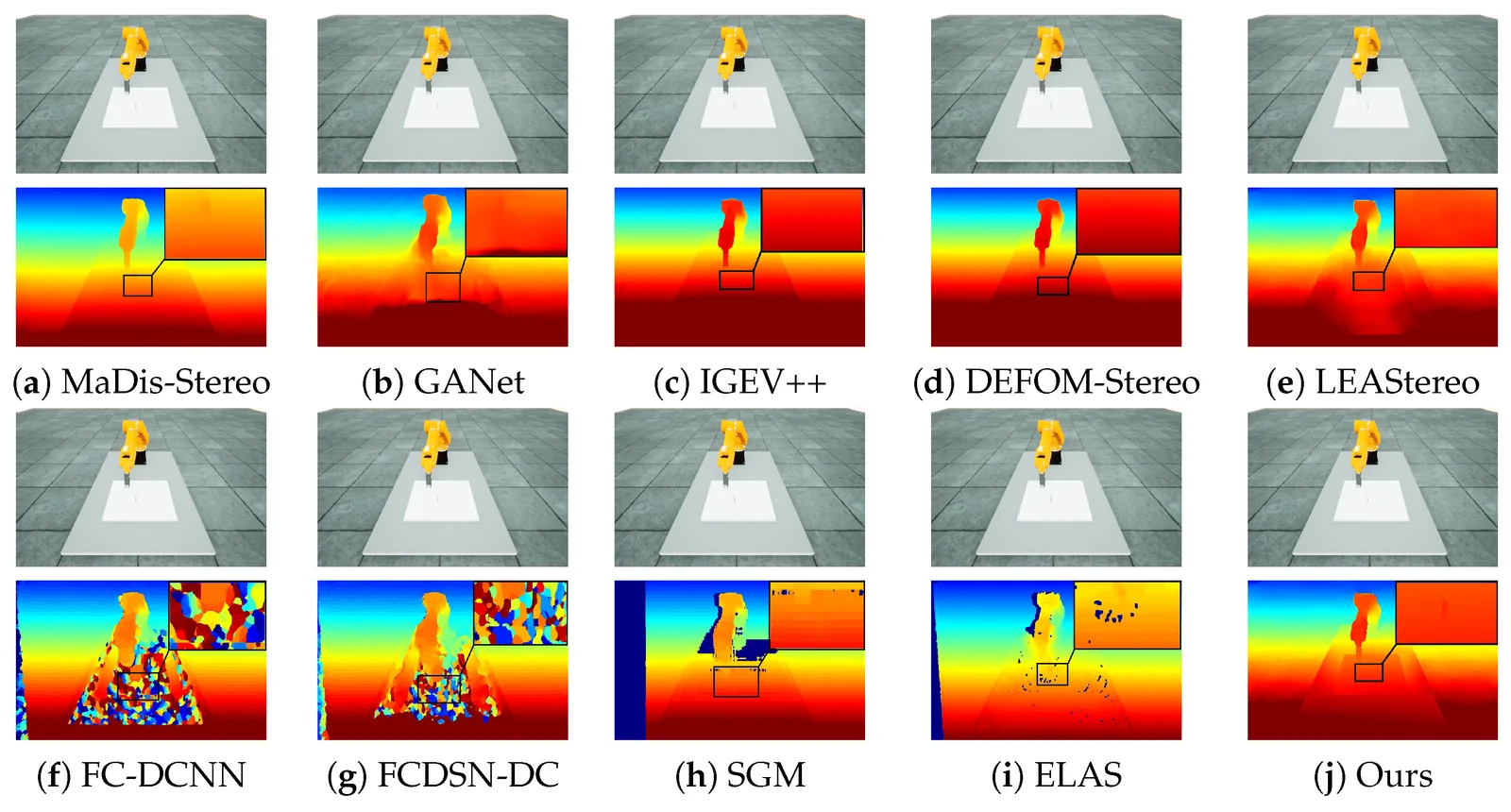
Qualitative comparison of disparity maps on the synthetic needle image (data1) across different algorithms. (**a**–**j**) Disparity maps of the ten compared methods on the synthetic needle scene.

**Qualitative Comparison on Synthetic Needle Image:** To further verify, at the visual level, how different algorithms perform on needle-containing images, Figure 14 presents a qualitative comparison of the disparity maps produced by ten algorithms on a needle image from the synthetic UE4 dataset. A consistent pattern emerges: although several algorithms reconstruct the overall scene reasonably well, all compared methods fail completely on the tiny needle region: the needle tip either vanishes or undergoes severe distortion. Only the proposed method preserves the full geometric structure of the needle.

**Figure 15:**
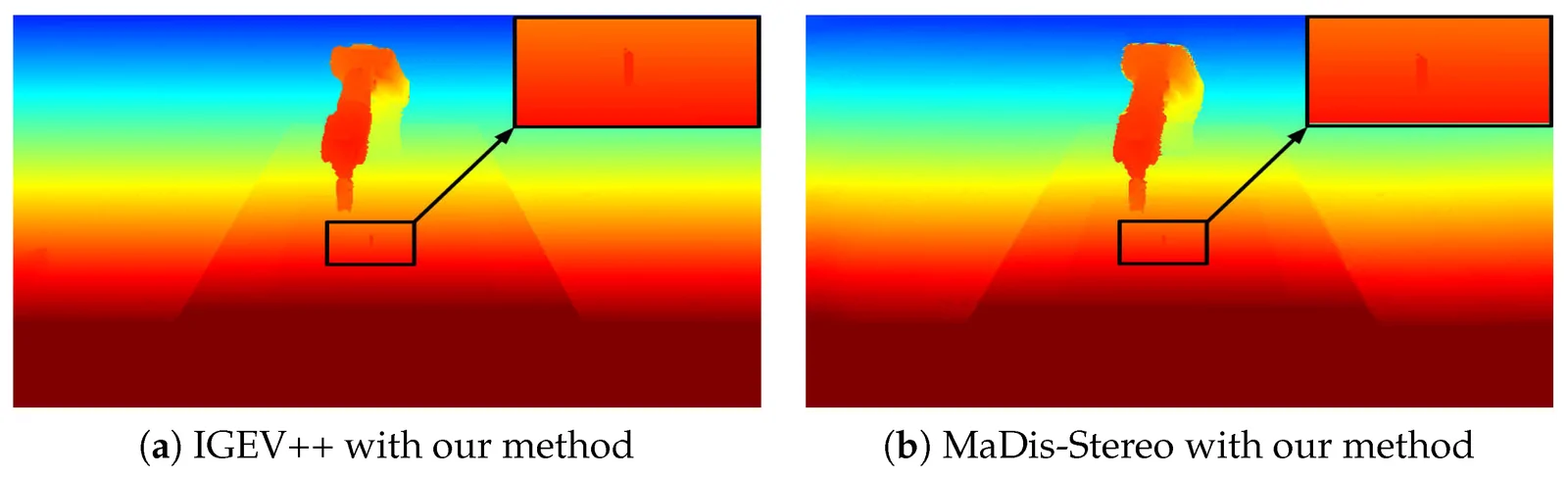
Results of overlaying the proposed method on the raw outputs of IGEV++ and MaDis-Stereo for the synthetic needle image. (**a**) IGEV++ with our method; (**b**) MaDis-Stereo with our method. The needle region is fully recovered after overlay in both cases.

**Post-Processing Overlay Verification:** To further verify the plug-and-play nature of our method, Figure 15 shows the result of directly overlaying the measurement refinement on the raw outputs of IGEV++ and MaDis-Stereo. Before overlay, both methods’ disparity degrades severely in the needle region: the needle tip is completely invisible. After overlay, the geometric structure of the needle is fully recovered. This directly demonstrates that the proposed method, without any network modification or retraining, can act as a universal post-processing module that enhances deep stereo methods’ measurement capability on tiny critical structures.

**Needle Region Quantitative Evaluation:** Table 6 presents a per-pixel quantitative evaluation of the needle region (31 columns × 21 rows, approximately 0.118% of the full image) in the synthetic dataset. Two complementary metrics are used for cross-validation: row-gradient narrow-peak detection (measuring whether the needle’s sharp-peak shape is preserved) and unified-background-threshold recall–precision (measuring needle-pixel detection accuracy). The proposed method is the **only one satisfying both criteria simultaneously**: row-gradient detection succeeds in 20/21 rows (the sharp-peak shape of the needle tip is fully preserved), and precision reaches 51.5%, more than double the next-best method, ELAS, at 21.1%. The other deep learning methods fall into two extremes: LEAStereo, SGM, GANet, IGEV++, FC-DCNN, and FCDSN-DC all achieve 0% recall (the needle tip is completely invisible); MaDis-Stereo reaches 93.5% recall but at the cost of severe expansion (410 false-positive pixels, precision only 9.5%); DEFOM-Stereo similarly trades 315 false detections for 21.7% recall. These results expose a general failure mode of deep stereo methods on tiny objects embedded in large planar regions: they either lose the target entirely or recover it only at the cost of severe expansion.

#### 4.2.2. Real Industrial Robot Scene

**Figure 16:**
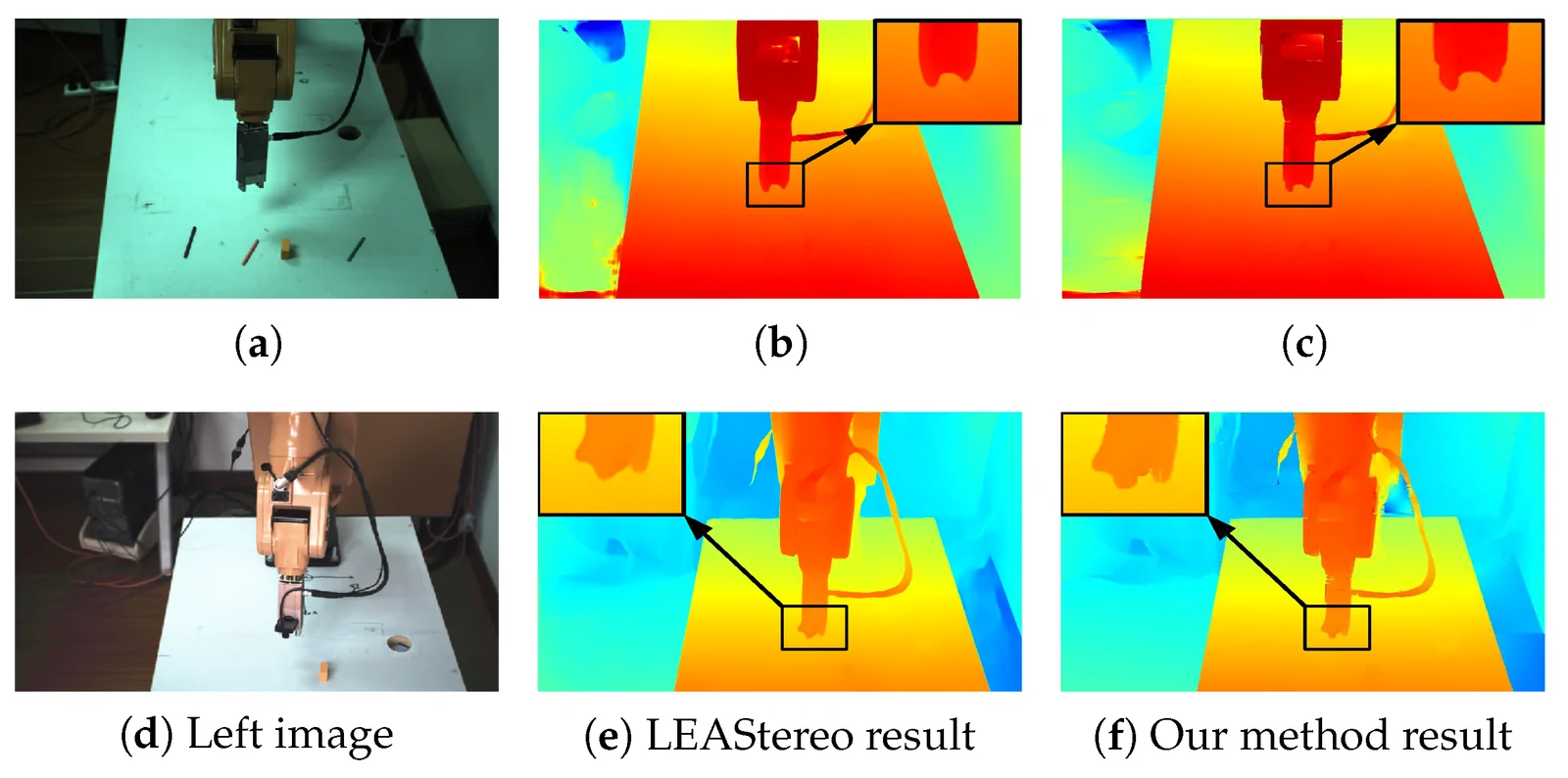
Industrial scene comparison: LEAStereo baseline vs. the proposed measurement refinement method. (**a**) Left image; (**b**) LEAStereo result; (**c**) Our method result; (**d**) Left image; (**e**) LEAStereo result; (**f**) Our method result.

**Industrial Scene Measurement Results:** We now evaluate the measurement performance on a real industrial robot scene. Figure 16 shows the processing results of the baseline LEAStereo method and of our measurement refinement method. The refined disparity is visibly more accurate, especially in the region most critical for robot guidance.

**Figure 17:**
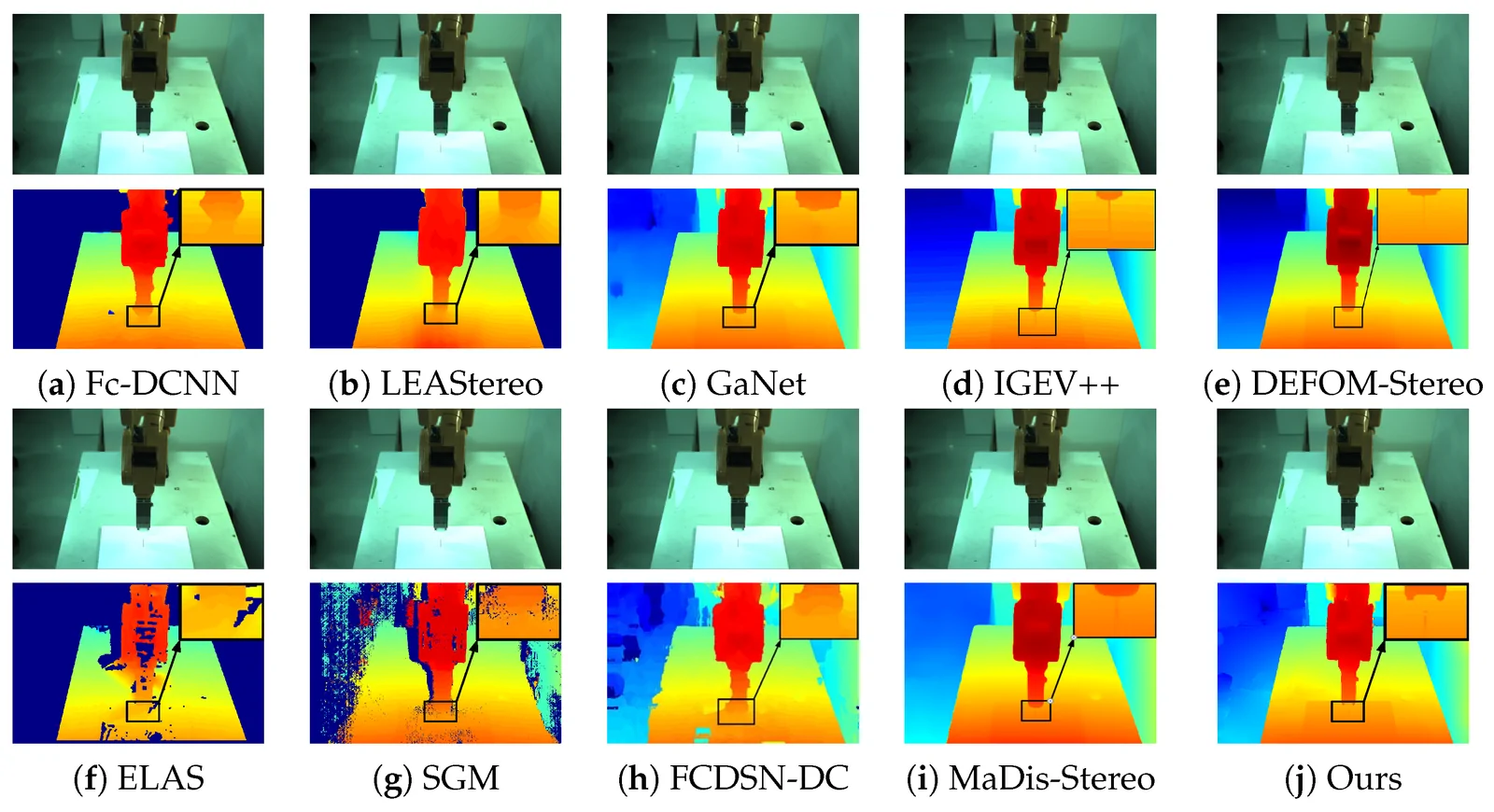
Industrial robot scene processing results. (**a**–**j**) Processing results of the ten compared methods on the real industrial needle scene.

Figure 17 lists the results of different methods on the needle scene. FC-DCNN, LEAStereo, MaDis-Stereo, and GANet all show clear disparity expansion; ELAS and SGM suffer from both voids and expansion. IGEV++ and DEFOM-Stereo, which lead the field, reconstruct the whole scene more strongly thanks to large-scale pretrained features. Yet at the boundary between the needle and the thin wire, both methods show disparity adhesion: the true physical gap disappears entirely. This repeats the finding from the Introduction: higher texture sensitivity does not equal better boundary measurement accuracy. The proposed method resolves these issues and produces high-precision disparity maps.

### 4.3. Middlebury Evaluation

**Figure 18:**
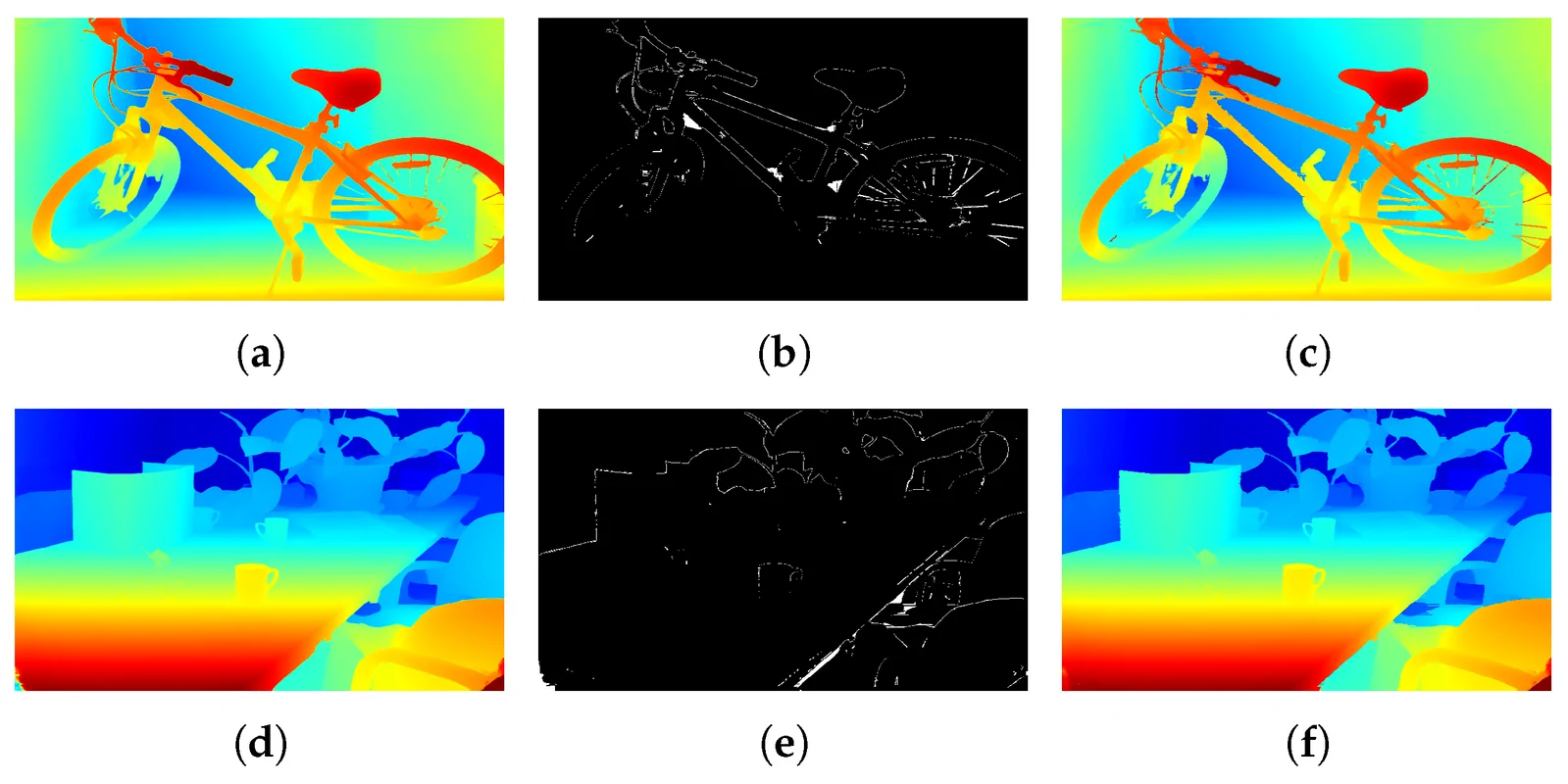
Disparity measurement refinement on Middlebury test images. (**a**–**c**) image bicycle2: (**a**) Original disparity map, (**b**) Disparity correction position, (**c**) Final disparity map; (**d**–**f**) image crusade: (**d**) Original disparity map, (**e**) Disparity correction position, (**f**) Final disparity map.

We use the Middlebury test set [21] (15 images) as our primary evaluation benchmark, because its structured-light ground truth is free of interpolation-based boundary inflation. Table 7 reports the results on all 15 test images, and Figure 18 shows the processed outputs for two representative images. The accuracy of the corrected disparity is verified by back-projecting the disparity values to the left and right image pair. The proposed measurement refinement method targets detail-level measurement accuracy. While the reduction in deviation rates may appear numerically modest, these fine-grained deviations are fundamentally difficult to address through deep learning alone. Correcting them becomes critical when high-precision depth measurement is required in real industrial scenarios. Since all 15 per-scene values are listed in full in Table 7, the scene-to-scene dispersion is directly visible: for the proposed method, the deviation rate spans 2.96–13.5% across the 15 scenes (mean 6.55%, with a standard deviation of 2.79 pp), tracking the LEAStereo baseline scene by scene with a per-scene difference from the baseline of at most 0.1 pp. The refinement therefore preserves the scene-level error profile of the baseline while applying a small, consistent correction on every scene.

## 5. Discussion

Relation to classical filtering. A uniform filter applies the same smoothing to every pixel and therefore cannot be selective; as noted in the Introduction, it suppresses noise but also erases thin targets. Our method instead follows geometric constraints and repairs the specific deviation types identified in this work, namely edge ownership errors, expansion spill-over, and submerged small objects, while leaving already correct regions untouched. The KITTI experiment exposes this selectivity directly: on seven deep networks the refinement changes the inflated-GT metric by only +0.35 to +0.60 pp, whereas on ELAS and SGM, whose outputs contain large voids and expansion, D1-all drops by 26.84 and 15.65 pp.

Relation to learned refinement. Iterative refinement modules such as those of IGEV++ and DEFOM-Stereo improve matching inside the network. Section 3.4, however, shows that fine-tuning on synthetic data does not recover submerged thin structures, because the limitation lies in the end-to-end architecture rather than in data quantity. Our method is complementary in this respect: it runs after any stereo method and restores the small structures that learning-based refinement leaves behind.

Metrological perspective. Section 2.3 shows that, when the focal length and baseline are exactly known, the depth uncertainty reduces to the disparity uncertainty through $\sigma_{D}/D\approx \sigma_{d}/d$. Section 4.2 confirms this link at the level of per-method results: refinement leaves the disparity error of already accurate deep methods essentially unchanged and markedly reduces that of traditional methods, so the depth uncertainty follows the same trend. This connects an accuracy metric such as D1 or EPE with a metrological one, namely measurement uncertainty, which is the quantity that a calibrated industrial measurement actually reports.

Limitations. The present industrial validation is qualitative, because pixel-level ground truth is unavailable in a real robotic cell. The quantitative claims rest on the synthetic UE4 dataset and on Middlebury. Extending the evaluation to task-level verification, for example needle-tip localization in real robot guidance, is left for future work, as is a systematic parameter-sweep sensitivity analysis of the three thresholds (the per-image variability analyses in Section 4.1 provide indirect evidence of robustness).

## 6. Conclusions

This paper analyzed the typical measurement errors in binocular stereo vision matching from a metrological perspective and proposed an error-aware measurement refinement method. First, we verified experimentally that the four systematic deviation classes shown by deep stereo matching methods are consistent across all tested architectures. The deviations persist even with clean ground truth (Middlebury and our UE4 dataset), and the edge inflation in KITTI LiDAR ground truth amplifies rather than causes them; our geometric-constraint-driven correction is unaffected by this inflation. Second, we proposed a four-module, error-aware measurement refinement method, with edge correction, expansion suppression, small-object matching, and warping removal. Each module follows a metrological diagnostic finding rather than an empirical heuristic. Third, we established a validation workflow that connects synthetic data generation, GUM [25] uncertainty analysis, and geometric-constraint-driven refinement. The workflow offers a template for developing measurement methods in scenarios where real ground truth is unavailable.

The UE4 synthetic industrial dataset used in this study has limitations as well. The gap between virtual rendering and the real physical world, in lighting, reflections, and microscopic material textures, cannot be fully eliminated. The scene layout is relatively simple, with random object placement on a tabletop, and the dataset is small (100 pairs, with only 10 used for evaluation). The dataset should be read as a controllable validation starting point that provides geometrically exact ground truth, not as a complete substitute for a real factory environment.

The results on real industrial robot images (Figure 16) are presented as qualitative evidence, because pixel-level ground truth cannot be obtained in physical industrial scenes without specialized measurement equipment (for example, coordinate measuring machines or structured-light scanners) that is incompatible with normal robot operation. The quantitative performance of the proposed method is validated on the UE4 synthetic industrial dataset (geometrically exact ground truth, Table 5) and on the Middlebury benchmark (structured-light ground truth, Table 7). Future work will explore task-level verification with calibration targets and multi-frame temporal stability analysis.

The limitations of the proposed method stem from its core design principle, geometric-constraint-driven processing. When geometric boundaries are unreliable (for example, foam whose color matches the tabletop, which makes the edge disappear, or wires whose pixel width falls below the detection threshold), the method conservatively produces no output rather than generating unreliable disparity values. From a metrological perspective, this is a principled strategy under uncertainty: an unreliable measurement hurts precision applications more than no measurement. From a scene-coverage perspective, this limitation is complementary to the strengths of texture-sensitive deep methods such as IGEV++ and DEFOM-Stereo, which cover more structures thanks to large-scale pretrained features, but pay for it with boundary adhesion and disparity expansion. Future work will explore fusing geometric constraints with semantic priors, aiming to extend recovery to low-contrast fine targets while preserving boundary accuracy, and to increase the scene diversity of the dataset.

## Figures and Tables

**Table 1:** State-of-the-art stereo matching methods and their reported accuracy on the KITTI 2015 benchmark (D1-all, all pixels, as reported in the respective papers).

Method	Venue	KITTI 2015 D1-All	Technical Approach
GANet [8]	CVPR 2019	1.93%	Guided cost aggregation
LEAStereo [9]	NeurIPS 2020	1.65%	Neural architecture search
IGEV++ [10]	TPAMI 2025	1.43% *	Geometry encoding volumes
DEFOM-Stereo [18]	CVPR 2025	1.41%	Foundation model transfer

* IGEV++ achieves top-tier accuracy on the KITTI 2015 benchmark; the D1-all value 1.43% is listed on the official KITTI leaderboard. As reported in its paper, it surpasses GMStereo by 14.69% on D1-all. The D1-all values in this table are taken from the respective papers and may follow different training protocols.

**Table 2:** Specifications of the UE4 synthetic industrial binocular dataset.

Parameter	Value	Note
Image pairs	100	10 pairs uniformly sampled for evaluation
Resolution	Multiple	Evaluation at 955×579 px
Focal length *f*	336 px	UE4 CameraActor
Baseline *B*	80 mm	CameraActor spacing
Working distance	≈1.20 m	Camera-to-needle distance
GT disparity	Geometric rendering	No interpolation/no occlusion hole-filling

**Table 3:** GUM depth measurement uncertainty budget.

Source	Type	Std. Value	Contrib. (mm)
Disparity $\sigma_{d}$	Type A	2.131 px	114.2
Combined $u_{c}\left(D\right)$	—	—	114.2
Expanded $U_{D}$ (k=2)	—	—	228.3

**Table 4:** Average quantitative evaluation on 10 images from the KITTI 2015 training set. B = Baseline, O = Ours (after measurement refinement), Δ = O − B. All seven deep learning methods show consistent degradation, indicating boundary GT inflation; traditional methods show substantial improvement.

Method	Type	B-D1	O-D1	ΔD1	B-Bad1	O-Bad1	ΔBad1	B-Bad4	O-Bad4	ΔBad4	B-EPE	O-EPE	ΔEPE
		(%)	(%)	(pp)	(%)	(%)	(pp)	(%)	(%)	(pp)	(px)	(px)	(px)
GANet	Deep	0.34	0.70	+0.36	3.52	3.94	+0.42	0.24	0.54	+0.30	0.322	0.352	+0.030
IGEV++	Deep	0.35	0.82	+0.47	2.60	3.16	+0.55	0.25	0.67	+0.41	0.273	0.313	+0.040
DEFOM	Deep	0.45	0.89	+0.44	3.12	3.64	+0.52	0.33	0.70	+0.37	0.297	0.334	+0.037
LEAStereo	Deep	1.03	1.38	+0.35	7.70	8.09	+0.39	0.76	1.06	+0.29	0.466	0.493	+0.028
MaDis-Stereo	Deep	1.31	1.67	+0.36	6.60	7.01	+0.42	1.01	1.31	+0.29	0.455	0.482	+0.027
FC-DCNN	Deep	5.46	5.90	+0.44	35.8	36.2	+0.40	4.10	4.53	+0.42	1.347	1.390	+0.043
FCDSN-DC	Deep	8.84	9.44	+0.60	43.5	44.0	+0.54	6.42	6.95	+0.53	1.884	1.934	+0.050
ELAS	Traditional	53.86	27.02	−26.84	67.7	52.7	−15.0	53.06	24.84	−28.22	19.71	7.098	−12.61
SGM	Traditional	34.90	19.25	−15.65	57.2	49.9	−7.26	33.50	16.31	−17.19	11.53	4.002	−7.53

**Table 5:** Quantitative evaluation on the synthetic UE4 dataset (10 images, dense GT, no inflation). B = Baseline, O = Ours, Δ = O − B.

Method	Bad1-B	Bad4-B	EPE-B	D1-B	Bad1-O	Bad4-O	EPE-O	D1-O	ΔBad1	ΔBad4	ΔEPE	ΔD1
	(%)	(%)	(px)	(%)	(%)	(%)	(px)	(%)	(pp)	(pp)	(px)	(pp)
MaDis	51.2	32.5	4.77	33.6	51.2	32.5	4.76	33.5	−0.01	−0.03	−0.01	−0.02
GANet	44.5	31.9	4.59	33.4	44.5	31.9	4.58	33.3	−0.01	−0.03	−0.01	−0.02
IGEV++	44.7	33.1	5.76	34.3	44.8	33.1	5.77	34.4	+0.12	+0.04	+0.01	+0.06
LEAStereo	56.8	44.4	6.88	46.8	56.9	44.5	6.89	46.8	+0.06	+0.05	+0.01	+0.06
DEFOM	51.6	33.4	7.14	35.4	51.6	33.3	7.13	35.4	−0.03	−0.08	−0.01	−0.06
FC-DCNN	56.4	39.6	8.65	41.5	56.4	39.6	8.63	41.5	−0.01	−0.02	−0.02	−0.03
FCDSN-DC	94.8	91.7	13.8	92.3	94.8	91.7	13.8	92.4	+0.02	+0.04	+0.01	+0.03
SGM	66.3	58.5	21.0	59.4	64.7	53.5	17.2	54.9	−1.63	−4.98	−3.80	−4.57
ELAS	66.6	59.5	21.4	60.3	64.5	53.5	16.9	54.8	−2.10	−5.98	−4.52	−5.52

**Table 6:** Per-pixel quantitative evaluation of the needle region (unified background threshold + row-gradient narrow-peak detection, cross-validated). The needle region accounts for only ∼0.118% of the full image.

Method	Row Gradient (21 Rows) Narrow-Peak Detection	TP	False Pos. (Expansion)	Recall (%)	Precision (%)
Our	20	35	33	76.1	51.5
LEAStereo	5	0	0	0.0	—
ELAS	21	46	172	100.0	21.1
SGM	0	0	0	0.0	—
GANet	10	0	0	0.0	—
IGEV++	4	0	112	0.0	—
DEFOM-Stereo	1	10	315	21.7	3.1
FC-DCNN	0	0	0	0.0	—
FCDSN-DC	0	0	0	0.0	—
MaDis-Stereo	0	43	410	93.5	9.5

Note: “—” in the Precision column indicates that the method detected zero pixels in the needle region (TP = 0, FP = 0), rendering precision mathematically undefined, as distinct from 0% precision, which means pixels were detected but all were false positives.

**Table 7:** Bad 4.0 disparity deviation rate on Middlebury test set (15 images).

Bad 4.0	Australia	AustraliaP	Bicycle2	Classroom2	Classroom2E	Computer	Crusade	CrusadeP	Djembe	DjembeL	Hoops	Livingroom	Newkuba	Plants	Staircase
DEFOM-Stereo	2.92	2.65	1.97	1.48	4.06	0.93	3.45	2.82	1.73	2.47	9.97	2.10	4.70	5.47	1.54
MaDis-Stereo	3.51	3.20	1.58	10.4	11.5	6.86	23.2	24.6	1.93	6.42	18.2	6.62	11.7	3.73	7.79
IGEV++	3.20	2.71	3.86	4.46	8.53	4.84	5.59	5.23	1.85	2.80	11.6	7.17	5.65	4.17	4.00
LEAStereo	4.59	3.55	3.16	5.63	7.77	8.46	5.86	4.77	2.97	5.94	13.6	9.11	9.13	7.14	6.83
Ga-Net	8.70	5.70	5.66	11.8	16.6	17.1	36.4	36.8	4.01	21.9	17.9	15.6	16.0	11.7	22.0
Fc-dcnn	14.9	6.20	11.2	17.2	32.3	28.8	15.2	15.4	6.25	23.9	27.5	18.6	20.9	17.2	24.5
FCDSN-DC	15.4	6.17	10.7	24.9	40.2	22.7	17.8	14.9	6.24	19.7	27.9	19.6	22.5	15.7	21.4
SGM	27.3	5.44	8.73	28.2	41.1	18.7	18.8	14.1	5.37	22.1	30.0	21.4	20.9	16.0	29.8
ELAS	29.9	11.5	13.4	25.0	66.1	26.9	28.0	21.9	9.49	36.6	38.4	25.3	29.1	34.0	29.2
Ours	4.58	3.54	3.15	5.62	7.77	8.45	5.85	4.75	2.96	5.94	13.5	9.10	9.12	7.13	6.82

## Data Availability

The KITTI 2015 and Middlebury datasets are publicly available. The synthetic UE4 dataset generated in this study is available from the corresponding author upon reasonable request.

## Abbreviations

GUM: Guide to the Expression of Uncertainty in Measurement; VIM: International Vocabulary of Metrology; SURF: Speeded-Up Robust Features; D1: percentage of erroneous pixels with disparity error larger than 1 pixel; EPE: End-Point Error; CNN: Convolutional Neural Network; NAS: Neural Architecture Search; GT: Ground Truth; UE4: Unreal Engine 4.
